# Sarcopenia in breast cancer: prognostic values and emerging therapeutic strategies

**DOI:** 10.3389/fsurg.2026.1884147

**Published:** 2026-07-15

**Authors:** Bo Xiang, Dongyi Zhao, Tao Liu, Guiying Xu

**Affiliations:** Department of Breast Surgery, Jilin Cancer Hospital, Changchun, China

**Keywords:** body composition, breast cancer, prehabilitation, sarcopenia, skeletal muscle

## Abstract

Breast cancer remains the most commonly diagnosed malignancy in women, yet treatment tolerance and long-term outcomes remain heterogeneous. Sarcopenia, a syndrome of progressive skeletal muscle loss, has emerged as a critical host factor in breast cancer, with prevalence ranging from 12% to 49% across the perioperative and neoadjuvant settings. Low muscle mass robustly predicts inferior overall and disease-free survival, reduced pathological complete response rates, heightened chemotherapy toxicity, and increased postoperative morbidity, particularly following complex autologous reconstruction. Integrating muscle metrics with systemic inflammatory indices further refines prognostic discrimination, while myosteatosis identifies patients with preserved muscle bulk but compromised metabolic fitness. Importantly, sarcopenia is potentially modifiable. Multimodal interventions centered on resistance training, protein optimization, and oral nutritional support show preliminary efficacy in preserving lean mass and attenuating treatment-related adverse effects. Emerging pharmacological strategies targeting the GDF-15–GFRAL and myostatin axes, alongside repurposed agents such as pindolol and sulfasalazine, offer promise for refractory cancer cachexia. Nevertheless, diagnostic heterogeneity and a paucity of breast cancer–specific randomized evidence continue to impede clinical translation. Standardized perioperative assessment, prospective prehabilitation trials, and scalable screening cascades are urgently needed to elevate muscle health from a passive biomarker to an active therapeutic target.

## Introduction

Breast cancer remains the most commonly diagnosed malignancy and the second leading cause of cancer-related death among women worldwide (1, 2). Despite decades of advances in population-based screening, systemic therapy, and precision oncology, approximately 20%–30% of patients with early-stage disease eventually develop distant recurrences, and a substantial proportion experience significant treatment-related morbidity (3–5). The majority of breast cancers are diagnosed at a curable stage, for which surgery constitutes the cornerstone of management, followed by adjuvant or neoadjuvant systemic therapy and, in selected cases, radiotherapy (6). In this evolving therapeutic landscape, traditional prognostic indices based solely on tumor stage, histology, and molecular subtype are increasingly recognized as incomplete; host factors, particularly body composition, have emerged as critical determinants of treatment tolerance and long-term outcomes (7, 8).

Sarcopenia, defined as a progressive, age-related syndrome characterized by the loss of skeletal muscle mass, strength, and physical performance, has traditionally been viewed as a geriatric condition (9). However, its prevalence and severity are markedly amplified in the oncological setting, where malignancy and its treatments impose a hypercatabolic state, systemic inflammation, and nutritional deficits that accelerate muscle wasting (10–12). In patients with solid tumors, sarcopenia represents not only a hallmark of cancer cachexia but also an objective, quantifiable measure of frailty and functional reserve, readily assessable via cross-sectional imaging such as computed tomography (CT), magnetic resonance imaging (MRI), dual-energy x-ray absorptiometry (DEXA), or bioelectrical impedance analysis (BIA) (13–15). Importantly, unlike many irreversible cancer biomarkers, sarcopenia is potentially modifiable, rendering it an attractive target for prehabilitation and supportive care (16–18).

In the specific context of breast cancer, accumulating evidence demonstrates that sarcopenia develops or worsens in the postoperative period, driven by surgical trauma, perioperative inflammatory cascades, reduced physical activity, and the subsequent cytotoxicity of chemotherapy and radiotherapy (19, 20). Retrospective and prospective studies have consistently shown that sarcopenic patients experience higher rates of postoperative complications, including infection, delayed wound healing, and prolonged hospitalization, as well as reduced tolerance to adjuvant chemotherapy (21, 22). The biological underpinnings of this association are multifactorial, encompassing tumour-derived and treatment-induced systemic inflammation, activation of the ubiquitin–proteasome and autophagy–lysosomal protein degradation pathways, mitochondrial dysfunction and oxidative stress, and endoplasmic reticulum stress responses that collectively drive skeletal muscle deconditioning and metabolic dysregulation (17, 23–25).

The clinical impact of sarcopenia varies significantly across breast cancer molecular subtypes. Recent studies demonstrate that while sarcopenia may not alter survival in luminal subtypes, it is a strong independent predictor of poor overall and disease-free survival in aggressive phenotypes, particularly triple-negative breast cancer (TNBC) and HER2-positive disease (26, 27). In the neoadjuvant setting, pretreatment sarcopenia is associated with significantly lower pathological complete response (pCR) rates and heightened chemotherapy toxicity in these subtypes (28). Furthermore, integrating skeletal muscle assessment with MRI-based radiomics has shown promise in further improving the predictive accuracy of neoadjuvant treatment efficacy in TNBC (29), highlighting the urgent need for subtype-specific risk stratification.

Despite the growing recognition of postoperative sarcopenia as a clinically relevant entity in breast cancer, several critical gaps persist. Diagnostic criteria and assessment modalities remain heterogeneous across studies, limiting cross-trial comparability. The precise molecular crosstalk between breast cancer biology and muscle homeostasis has yet to be fully elucidated. Furthermore, although multimodal interventions combining nutritional optimization and structured exercise have shown preliminary efficacy in improving muscle mass and reducing complications, robust, prospective randomized evidence guiding standardized clinical practice is still lacking. In this Review, we synthesize the current epidemiological, mechanistic, and interventional evidence to define postoperative sarcopenia in breast cancer, evaluate its prognostic value, dissect the underlying pathophysiological pathways, and discuss emerging strategies for prevention and treatment.

## Definition and prevalence of postoperative sarcopenia

Sarcopenia is formally recognized as a progressive, age-related syndrome characterized by the generalized loss of skeletal muscle mass, strength, and physical performance (30). Although originally conceptualized within geriatric medicine, its diagnostic framework has been refined through successive international consensus statements. The European Working Group on Sarcopenia in Older People 2 (EWGSOP2) established a three-axis algorithm requiring low muscle strength, low muscle quantity or quality, and reduced physical performance, with severity stratified according to the number of criteria fulfilled (31). More recently, the Global Leadership Initiative in Sarcopenia (GLIS) and the Asian Working Group for Sarcopenia (AWGS) 2025 consensus have streamlined this approach, defining sarcopenia as the concurrent presence of low muscle mass and low muscle strength, while repositioning physical performance measures as downstream outcome indicators rather than diagnostic prerequisites (32).

In oncology, and specifically within the context of breast cancer, these definitions require careful contextual adaptation. Cancer-associated sarcopenia diverges from the classic geriatric phenotype because tumor biology and anticancer therapies, including surgery, chemotherapy, and radiotherapy, impose distinct hypercatabolic and proinflammatory pressures that accelerate muscle wasting beyond age-related physiological decline (33–35). While the terms sarcopenia and cachexia are frequently used interchangeably in the oncological literature, distinguishing between them is critical for defining therapeutic windows (36). Cachexia represents a complex, multi-organ metabolic syndrome driven by severe systemic inflammation, characterized by involuntary weight loss involving both adipose and muscle tissue, and is largely refractory to conventional nutritional support in its advanced stages (37). In contrast, sarcopenia specifically denotes the depletion of skeletal muscle mass and function (37). This distinction is particularly relevant in breast cancer, where many patients, especially those undergoing neoadjuvant or adjuvant therapies, experience profound skeletal muscle loss independent of overt clinical weight loss, frequently in the context of preserved or increased adiposity (38–40). Therefore, we utilize the term “sarcopenia” throughout this review to emphasize these early, muscle-specific morphological and functional changes. Unlike end-stage cancer cachexia, cancer-associated sarcopenia is a quantifiable, targetable entity that often responds to timely exercise and metabolic interventions.

Oncology research has historically relied heavily on objective, imaging-based quantification of muscle mass, most commonly using cross-sectional computed tomography (CT) at the third lumbar vertebra (L3) level or magnetic resonance imaging (MRI), with sex-specific thresholds for skeletal muscle index (SMI) (41). Dual-energy x-ray absorptiometry (DXA) and bioelectrical impedance analysis (BIA) provide alternative estimates of appendicular lean mass; however, their utility in the postoperative breast cancer setting may be confounded by treatment-related fluid shifts, lymphedema, or altered hydration status (42, 43). The AWGS 2025 update further introduces BMI-adjusted cutoffs and extends validated diagnostic thresholds to middle-aged adults, an expansion particularly relevant to breast cancer populations where a substantial proportion of patients are premenopausal or perimenopausal (32).

Reported prevalence estimates of sarcopenia in breast cancer vary widely across the perioperative and oncological continuum, reflecting heterogeneity in diagnostic thresholds, imaging modalities, baseline demographics, and treatment contexts (Table 1). Among 88 Japanese patients undergoing total mastectomy, Yabe et al. documented sarcopenia in 49% when applying the psoas muscle index derived from preoperative computed tomography (44). In the neoadjuvant chemotherapy (NAC) setting, baseline rates diverge markedly by geography and cohort characteristics. Butler et al. reported a sarcopenia prevalence of 12.2% among 258 patients (mean age 49.5 years, BMI 27.6 kg/m^2^) at a Western tertiary referral center, defining sarcopenia as a skeletal muscle index <38.5 cm^2^/m^2^ at the L3 vertebral level on axial CT (45). By contrast, East Asian cohorts have consistently reported higher baseline burdens. In a retrospective series of 317 Korean women with breast cancer scheduled for NAC, Jang et al. identified sarcopenia in 25.2% prior to treatment initiation, and longitudinal assessment showed that 80% of those sarcopenic at baseline retained this status after chemotherapy completion (46). Regimen-specific vulnerability is also evident: in a related cohort of 298 Korean patients, those receiving TCHP (docetaxel, carboplatin, trastuzumab, and pertuzumab) developed new-onset sarcopenia at a rate of 12%, compared with only 1% among patients treated with AC-T (anthracycline and cyclophosphamide followed by taxane) (47). In a companion analysis of the same 298-patient Korean cohort, Jang et al. further demonstrated that sarcopenic patients experienced twice the rate of severe chemotherapy-induced anemia as their non-sarcopenic counterparts, underscoring the clinical imprint of baseline muscle depletion (48). Complementing these NAC data, Du et al. observed a sarcopenia prevalence of 29.8% (85 of 285) among Chinese patients with early-stage breast cancer undergoing chemotherapy, diagnosed via CT-based criteria (49). Zhang et al. further corroborated NAC-associated muscle wasting in a smaller Chinese cohort of 43 patients, documenting significant post-treatment reductions in pectoral muscle area and density, although a formal sarcopenia prevalence was not explicitly quantified (50). Extending into the post-mastectomy reconstructive phase, Hehir et al. performed a systematic review and meta-analysis of six studies comprising 1,315 patients undergoing autologous breast reconstruction and found that sarcopenic individuals accounted for approximately 31% of the pooled population (407 sarcopenic versus 908 non-sarcopenic), with muscle mass quantified via CT-derived psoas muscle index or skeletal muscle index (51). Collectively, these findings indicate that sarcopenia affects anywhere from approximately 12% to 49% of breast cancer patients depending on the clinical setting, ethnic background, and therapeutic exposure, underscoring the necessity for standardized, setting-specific surveillance across the preoperative and postoperative trajectory.

**Table 1:** Summary of studies on prevalence of sarcopenia in breast cancer.

Study (first author, year)	Population	Clinical context	Sample size (*n*)	Sarcopenia prevalence	Diagnostic method/key cohort characteristics
Butler et al., 2025 (45)	Ireland	Neoadjuvant chemotherapy (NAC) for breast cancer	258	12.2% (24/258)	SMI <38.5 cm^2^/m^2^ at L3 on axial CT
Hehir et al., 2025 (51)	Mixed	Autologous breast reconstruction after mastectomy	1,315	31.0% (407/1,315)	CT-derived PMI or SMI
Du et al., 2024 (49)	Chinese	Early-stage breast cancer receiving chemotherapy	285	29.8% (85/285)	CT-based diagnosis
Zhang et al., 2024 (50)	Chinese	NAC, body composition & metabolic analysis	43	Not reported	Pectoral muscle area/density on chest CT
Jang et al., 2022 (46)	Korean	NAC, longitudinal body composition change	317	25.2% (80/317)	SMI on abdominal CT
Jang et al., 2022 (47)	Korean	NAC, regimen comparison (AC-T vs. TCHP)	298	12% (TCHP) vs. 1% (AC-T)	SMI on abdominal CT
Yabe et al., 2021 (41)	Japanese	Preoperative total mastectomy	88	49% (43/88)	Psoas muscle index on preoperative CT

## Prognostic value of sarcopenia and related metric

Beyond its epidemiological footprint, sarcopenia has emerged as a robust, independent prognostic determinant across the breast cancer care continuum. Accumulating evidence demonstrates that low skeletal muscle mass consistently stratifies patients into distinct risk tiers for overall survival (OS), disease-free survival (DFS), pathological treatment response, perioperative morbidity, and chemotherapy tolerance (49, 52). In this section, we dissect the prognostic landscape of sarcopenia and related composite metrics in breast cancer, synthesizing imaging-based, serological, and causal inference evidence.

The association between skeletal muscle depletion and inferior long-term outcomes represents one of the most consistently replicated findings in breast cancer sarcopenia research. In a retrospective cohort of 577 Korean women with non-metastatic invasive breast cancer, Huh et al. demonstrated that low total abdominal muscle area (TAMA) measured on preoperative PET-CT was a strong independent prognostic biomarker for OS, with an even more pronounced effect in patients aged ≥50 years (53). Similarly, Du et al. reported that sarcopenic Chinese patients with early-stage breast cancer exhibited significantly reduced OS rates at 1 year (82.94% versus 85.78%), 3 years (81.76% versus 83.91%), and 5 years (80.59% versus 81.17%) compared with non-sarcopenic counterparts (*P* < 0.001) (49). In the neoadjuvant setting, Butler et al. found that lower lean body mass independently predicted reduced OS and invasive disease-free survival among 258 patients receiving neoadjuvant chemotherapy, although sarcopenia *per se* did not reach statistical significance for disease-specific survival (45). These data collectively establish muscle mass as a critical reservoir of host resilience, with its depletion heralding accelerated disease progression and mortality.

Emerging evidence further suggests that the integration of muscle metrics with systemic inflammatory indices amplifies prognostic discrimination beyond muscle mass alone. Tang et al. evaluated 97 patients with lymph node-positive breast cancer after radical mastectomy and demonstrated that a composite phenotype integrating skeletal muscle status with the systemic inflammation index (SII) yielded substantial prognostic power; specifically, the concomitant presence of adverse muscle and inflammatory profiles conferred a multiplicative risk for poor 5-year OS (HR 16.36; *P* = 0.016) compared with either parameter alone (54). Similarly, Hua et al. showed that combining skeletal muscle index (SMI) with the monocyte-to-lymphocyte ratio (MLR) independently predicted OS and recurrence-free survival in patients receiving postoperative adjuvant radiotherapy, reinforcing the concept that integrated muscle-inflammation biomarkers capture host-tumor interactions more comprehensively than isolated muscle metrics (55). These findings position the muscle-inflammation axis as a central hub in breast cancer prognostication and provide a rationale for multimodal risk scores that concurrently assess musculoskeletal and immune-inflammatory status.

In the neoadjuvant chemotherapy (NAC) paradigm, where pathological complete response (pCR) serves as a validated surrogate for long-term survival sarcopenia has emerged as a modifiable predictor of treatment efficacy. Günaltılı et al. analyzed 85 patients with HER2-positive or TNBC undergoing NAC and identified low SMI as the sole independent predictor of pCR on multivariate logistic regression; patients without low SMI had four-fold higher odds of achieving (28). Concordantly, Isıklar et al. reported that both sarcopenia and obesity independently and synergistically contributed to poorer pathological responses after NAC, with non-sarcopenic patients achieving a 62.5% pCR rate compared with 37.5% in sarcopenic individuals (56). However, conflicting data exist: Butler et al. observed no association between sarcopenia and pCR, nodal positivity after NAC, or Sataloff tumor response in a Western cohort of 258 patients (45). Such discrepancies likely reflect heterogeneity in sarcopenia thresholds, imaging protocols, ethnic differences in body composition, and varying chemotherapy backbones, underscoring the need for standardized, subtype-specific definitions.

Sarcopenia not only impairs oncological response but also erodes physiological reserve, predisposing patients to heightened treatment-related toxicity. In the NAC setting, Günaltılı et al. documented that low SMI was associated with a significantly higher rate of grade 3–4 treatment-related toxicity (28). Du et al. corroborated these observations by showing that sarcopenic patients randomized to multimodal interventions combining supervised exercise and tailored nutritional support experienced substantially reduced chemotherapy side effects and postoperative complication rates compared with controls (49). These data imply that sarcopenia-mediated toxicity arises from altered drug distribution volumes, decreased protein binding, and compromised organ functional reserve, all of which curtail dose intensity and therapeutic efficacy.

The prognostic impact of sarcopenia extends into the surgical domain, although its association with postoperative morbidity varies markedly by procedure complexity and measurement modality. In autologous microsurgical breast reconstruction, Pittelkow et al. reported that sarcopenic patients experienced significantly higher rates of flap-site delayed healing (37.5% versus 20.0%; *P* = 0.046), unplanned return to the operating room (25% versus 11.6%; *P* = 0.05), and prolonged ICU and hospital length of stay (*P* < 0.0005) (57). A subsequent systematic review and meta-analysis by Hehir et al. of six studies encompassing 1,315 patients confirmed that sarcopenia increased the risk of postoperative hematoma formation, although no significant differences were observed for total flap loss, recipient-site infection, seroma, or donor-site complications (51). By contrast, Broyles et al. found no association between sarcopenia and minor or major complications in abdominally based free-flap reconstruction following post-mastectomy radiotherapy (58), and Aleixo et al. similarly reported no correlation between sarcopenia and adverse surgical outcomes in a large cohort of 682 patients with stage I–III breast cancer, identifying obesity as the dominant risk factor for complications following mastectomy with reconstruction (52). Collectively, these findings suggest that sarcopenia's predictive value for surgical complications is context-dependent, being most pronounced in complex, multi-step reconstructive procedures where microvascular integrity and wound healing demand substantial nutritional and metabolic reserve.

The recognition that muscle quantity alone incompletely captures host vulnerability has catalyzed interest in composite and alternative metrics. Morikawa et al. demonstrated that the SMI-to-BMI ratio was a superior predictor of post-surgical bleeding after mastectomy compared with either SMI or BMI alone, and on multivariate analysis, a low ratio conferred a 38.4-fold increased hazard for bleeding (59). This paradigm shift toward normalized, body-size-adjusted indices addresses the limitations of absolute muscle thresholds in ethnically and metabolically diverse populations. Serological surrogates have also gained traction: Wang et al. introduced the serum creatinine   ×   cystatin C-based eGFR index (SCr   ×   eGFR[cys]) as a pragmatic biomarker for sarcopenia screening, demonstrating that higher quartiles were independently associated with favorable OS in non-metastatic breast cancer after surgery (60). Such indices offer readily accessible, preoperative risk stratification without requiring specialized imaging. Furthermore, Kumar et al. advanced the conceptual framework by distinguishing myosteatosis as a distinct, early, and targetable biomarker that reflects impaired muscle quality and metabolic dysfunction rather than mere mass depletion, appearing early in the trajectory of muscle deterioration before irreversible cachexia develops (61). Myosteatosis may thus identify a patient subset with preserved muscle bulk but compromised metabolic fitness, who would be misclassified by conventional SMI cutoffs.

While observational studies robustly associate sarcopenia with adverse breast cancer outcomes, disentangling correlation from causation remains methodologically challenging. Xu et al. addressed this limitation by leveraging two-sample Mendelian randomization (MR) alongside National Health and Nutrition Examination Survey (NHANES) data, providing causal evidence that appendicular lean mass (ALM) and whole-body lean mass (WBLM) exert protective, causal negative effects on overall cancer occurrence (19). Notably, sarcopenia defined by Foundation for the National Institutes of Health (FNIH) criteria significantly increased the risk of overall cancer types, with MR analysis confirming causal effects on lymphoid haematopoietic malignancies, lymphoid leukaemia, and breast cancer specifically. These genetic instrumental variable analyses strengthen the biological plausibility that sarcopenia is not merely a passive bystander reflecting advanced disease, but an active contributor to cancer pathogenesis and progression, likely mediated through chronic low-grade inflammation, insulin resistance, and altered immune surveillance (62–64).

## Potential therapeutic interventions on sarcopenia

### Screening and operational definitions

The clinical management of sarcopenia in breast cancer begins with unambiguous diagnostic criteria and feasible screening pathways. The 2025 consensus update from the Asian Working Group for Sarcopenia (AWGS) defines sarcopenia as the concurrent presence of low muscle mass and low muscle strength, deliberately repositioning physical performance measures as downstream outcome indicators rather than prerequisite diagnostic criteria (32). This streamlined, two-axis framework aligns closely with the Global Leadership Initiative on Sarcopenia (GLIS) conceptual model and facilitates pragmatic implementation in oncology settings where repeated functional testing may be impractical during active chemotherapy or postoperative recovery.

Recent advances have sought to overcome these barriers through anatomical surrogates, refined functional metrics, portable technologies, and composite biomarkers. Zare et al. demonstrated in a meta-analysis that cervical (C3) muscle quantification on routine CT exhibits a strong pooled correlation with the L3 reference, with even stronger agreement when sex- and BMI-specific thresholds are applied, principle highly relevant to breast cancer, where thoracic imaging is frequently obtained for staging and radiotherapy planning (65). Concurrently, Yin et al. operationalized the GLIS framework by proposing the lower-limb skeletal muscle mass to five-time chair stand test ratio (LFR) as a measure of muscle-specific strength, and showed that a definition combining low handgrip strength or low LFR outperformed traditional algorithms in predicting functional decline (66). On the screening front, the Sarco-Detect study confirmed that BIA phase angle and handgrip strength correlate significantly with CT-SMI, whereas the SARC-F questionnaire offers high specificity but low sensitivity, rendering it more suitable for ruling out sarcopenia than for case-finding (67). Wu et al. further highlighted the utility of calf circumference, bedside ultrasound, and self-administered tools such as the Yubi-wakka test for community-based triage (68), while Mehra et al. showed that wearable and smartphone-based technologies achieve correlations >0.80 against DXA, although they currently remain adjunctive monitors rather than diagnostic replacements (69). Finally, composite risk models are gaining traction: Xu et al. demonstrated that combining the alkaline phosphatase-to-prealbumin ratio with CT-quantified sarcopenia yields superior prognostic discrimination (70), and Jeong et al. developed a simplified clinical model using sex, age ≥60 years, extent of surgery, and body weight loss ≥8 kg that achieved an AUROC of 0.792 for identifying postoperative sarcopenia (71). Collectively, these innovations point toward a pragmatic, tiered screening cascade for breast cancer, initial triage via low-burden tools, followed by targeted imaging or BIA, and ultimately refined by composite metrics that embed muscle status within broader host-nutritional contexts.

### Exercise and lifestyle interventions

Physical activity represents the cornerstone of sarcopenia management across oncological populations, with accumulating evidence supporting its efficacy in preserving muscle mass, strength, and functional capacity during active cancer treatment (72). Bibliometric analyses reveal exponentially growing research interest in the intersection of cancer, sarcopenia, and physical activity over the past decade, with resistance exercise emerging as a dominant thematic cluster (43). A recent systematic review and meta-analysis of 59 randomized controlled trials (RCTs) demonstrated that sarcopenia interventions yield statistically significant improvements in muscle mass, muscle strength, and select measures of physical performance, with multi-component approaches conferring the greatest benefit (73).

Within the breast cancer continuum, resistance training (RT) has emerged as a particularly potent modality, yet remains underutilized in clinical practice. In the post-treatment recovery phase, RT rebuilds muscular strength, attenuates chemotherapy-induced sarcopenia, reduces fat accumulation, and improves lymphatic flow without exacerbating lymphedema, addressing long-held safety concerns that previously limited its adoption (74). Mechanistically, acute and chronic exercise partially interrupts the vicious cycle of muscle wasting by activating the mTOR pathway to enhance muscle protein synthesis while concurrently suppressing the expression of muscle atrophy-related genes, including MuRF-1 (TRIM-63) and ATROGIN-1 (FBXO32), thereby reducing ubiquitin-proteasome-mediated protein degradation (75). Furthermore, strength training modulates muscle-specific microRNAs (myomiRs), which regulate protein synthesis and may serve as early biomarkers of sarcopenia pathophysiology (76).

The neoadjuvant chemotherapy (NAC) window offers a critical opportunity for prehabilitative intervention. An ongoing RCT protocol specifically targets sarcopenia in breast cancer patients undergoing NAC through a supervised, hybrid resistance exercise program, with preliminary frameworks suggesting improvements in body composition, muscle strength, and treatment-related adverse effects (77). Prehabilitation models combining daily walking, resistance training, and nutritional supplementation have demonstrated efficacy in attenuating lean body mass loss prior to major abdominal surgery, supporting the biological plausibility of analogous approaches in the preoperative breast cancer setting (78). However, standardized definitions and outcome metrics for muscle wasting remain essential to evaluate prehabilitation efficacy across cancer types (79).

Beyond structured exercise programs, broader lifestyle modifications exert measurable effects on musculoskeletal health. Mendelian randomization analyses have provided causal evidence linking moderate-to-vigorous intensity physical activity during leisure time to reduced sarcopenia risk, whereas prolonged leisure screen time and smoking demonstrate deleterious causal relationships (80). In cancer populations, smoking duration and malnutrition synergistically increase mortality risk, with each additional five years of smoking increasing death risk by 6% among smokers, and by 86% per decade among malnourished patients, mediated in part by elevated systemic inflammation (81). Additionally, high-fat diet and pre-existing obesity independently exacerbate muscle wasting, tumor growth, and IL-6-mediated inflammatory signaling in experimental cachexia models, underscoring the importance of adiposity control and dietary quality alongside exercise prescription (82). Notably, meeting both vigorous and moderate physical activity guidelines in conjunction with higher protein intake is positively associated with appendicular lean soft tissue index in middle-aged adults with cancer, whereas either intervention alone shows no significant association, highlighting the potential necessity of combined lifestyle strategies (83).

### Nutritional interventions

Nutritional optimization constitutes an indispensable adjunct to exercise in countering cancer-related muscle wasting. As comprehensively reviewed in gastrointestinal malignancies, the pathological mechanisms of cancer-related sarcopenia encompass systemic inflammation, metabolic disorders, and treatment-related stress, all of which inform nutritional intervention design (33). Protein intake represents the primary nutritional determinant of muscle anabolism; a multicenter longitudinal cohort study of 5,079 Chinese cancer patients revealed that median total protein intake was 55.0 g/day, predominantly dietary, with red meat as the dominant animal protein source across all intake strata (84).

The timing, distribution, and route of protein delivery also warrant clinical attention. Oral nutritional supplements (ONS) are widely recommended to support inadequate oral intake during chemotherapy, although their metabolic effects vary by cachexia stage. In a single-arm pilot study of 10 patients with cancer cachexia undergoing chemotherapy, 8-week ONS intervention improved handgrip strength and walking speed, with metabolomics revealing differential fatty acid responses between severe and non-severe cachexia patients (85). Short-term ONS following hospital discharge has also been shown to attenuate postoperative skeletal muscle loss in gastric cancer patients and improve long-term prognosis, particularly among elderly patients, those with poor baseline nutritional status, and individuals undergoing total gastrectomy (86).

Amino acid supplementation has garnered particular interest as a strategy to correct cancer-associated protein imbalances. Targeted mixtures of essential amino acids (EAAs) have demonstrated capacity to improve muscle mass, strength, and quality of life in hypercatabolic conditions, with emerging *in vitro* and *in vivo* data suggesting potential inhibitory effects on tumor proliferation (87). However, concerns persist that increased availability of certain amino acids might stimulate tumor growth in specific contexts, rendering balanced EAA formulations, rather than single-amino-acid megadoses, a more prudent adjunctive strategy (87).

Systematic reviews of nutritional interventions in older adults with cancer confirm that protein supplementation notably improves lean body mass and skeletal muscle index in select RCTs, while observational studies associate high fish and fat intake with reduced sarcopenia risk (88). Nevertheless, significant heterogeneity in study designs, outcome measures, and intervention protocols limits the strength of current recommendations (88, 89). Whole-course nutrition management, encompassing preoperative, perioperative, and postoperative phases, has demonstrated efficacy in preserving skeletal muscle mass in gastric cancer patients undergoing neoadjuvant treatment, suggesting that continuous, multidisciplinary nutritional oversight may be superior to episodic supplementation (90). In breast cancer, multimodal interventions combining supervised exercise with tailored nutritional support have already shown preliminary efficacy in reducing chemotherapy side effects and postoperative complication rates (15), reinforcing the imperative for integrated, rather than siloed, supportive care.

### Novel therapeutic approaches

While lifestyle and nutritional interventions form the foundation of sarcopenia management, pharmacological strategies targeting specific molecular pathways are advancing rapidly, offering promise for patients who remain refractory to conservative measures or who face accelerated wasting during intensive oncological therapy.

The GDF-15–GFRAL axis has emerged as a tractable therapeutic target in cancer cachexia. In a recent Phase 2 trial, the anti-GDF-15 antibody ponsegromab increased body weight, appetite, muscle mass, and physical activity in cachectic patients (91). Preclinical studies further demonstrate that the efficacy of myostatin pathway inhibition is context-dependent on GDF-15 status: in mouse models with low circulating GDF-15, combining anti-myostatin and anti-GDF-15 antibodies produced synergistic gains in body weight and hindlimb muscle mass compared with monotherapy, whereas in high-GDF-15 models, anti-myostatin monotherapy was largely ineffective unless combined with GDF-15 neutralization (92). Beyond these mechanistic approaches, an antimyostatin peptibody prevented lean body mass loss (mean difference = 2.2 kg; SE 0.8%) in patients with advanced prostate cancer undergoing androgen deprivation therapy (89), confirming the cross-tumor applicability of myostatin-directed strategies.

Several repurposed pharmacological agents have shown preclinical promise. Pindolol, a non-selective *β*-adrenergic receptor antagonist with 5-HT1A partial agonist activity, mitigated cancer cachexia in a murine melanoma model by reducing systemic inflammation, preserving skeletal muscle and adipose depots, improving glucose levels and insulin sensitivity, and downregulating TRIM63 and Atrogin-1 expression (93). Sulfasalazine, an established anti-inflammatory agent used in inflammatory bowel disease, was identified through an FDA-approved drug library screen as an inhibitor of PHF20-induced YY1 transcriptional activity, which suppresses muscle differentiation; in mouse models of muscle atrophy, sulfasalazine enhanced muscle strength and function while mitigating muscle loss, and clinical data from IBD patients revealed significantly higher total psoas index among those treated with sulfasalazine (94). Additionally, oxytocin, an approved neuropeptide with declining expression during aging, restored skeletal muscle mass, fiber cross-sectional area, and overall body weight in C26 tumor-bearing mice while reducing MuRF1 and Atrogin1 expression by over sixfold; metabolic proteomics further demonstrated that oxytocin restored the synthesis of key proteins essential for muscle regeneration (95).

Selective androgen receptor modulators (SARMs), such as enobosarm and MK-0773, have also emerged as a potentially safer alternative to conventional androgenic steroids (96, 97). Phase II and III clinical trials have demonstrated that enobosarm significantly increases lean body mass (LBM) in patients with cancer-induced muscle wasting and in healthy older cohorts (98). However, the translation of SARMs into routine practice is currently impeded by a frequent clinical discordance: while these agents effectively induce muscle hypertrophy, these morphological gains rarely translate into statistically significant improvements in physical performance or muscle strength (99).

Another highly promising avenue involves the inhibition of negative muscle regulators, specifically targeting the myostatin and activin type II receptor (ActRII) signaling pathways (100). Myostatin, a transforming growth factor-*β* (TGF-*β*) superfamily member, accelerates muscle degradation via the ubiquitin-proteasome system (100). Monoclonal antibodies targeting ActRII, notably bimagrumab, have elicited profound hypertrophic responses (101, 102). Crucially, bimagrumab uniquely drives substantial reductions in adipose tissue mass alongside LBM expansion (103, 104). This dual-action metabolic profile presents an ideal therapeutic mechanism for addressing the sarcopenic obesity phenotype frequently observed in women experiencing metabolic and compositional shifts following breast cancer chemotherapy and endocrine therapy.

The profound impact of sex hormones on skeletal muscle homeostasis offers a critical lens for understanding sarcopenia. In postmenopausal women, lower endogenous free estradiol and testosterone, along with elevated sex hormone-binding globulin (SHBG), correlate with reduced lean mass and a higher risk of sarcopenia (105). While some observational data suggest menopausal hormone replacement therapy (HRT) preserves muscle, recent systematic reviews emphasize a lack of high-quality evidence demonstrating definitive functional benefits (106). In the breast cancer setting, where traditional HRT is contraindicated, the endocrine-sarcopenia axis remains highly relevant. For instance, baseline sarcopenia in women with early-stage hormone receptor-positive breast cancer independently predicts toxicity-related early discontinuation of adjuvant endocrine therapy (aET), leading to significantly worse disease-free survival (107).

The interplay between chronic low-grade inflammation and dysregulated energy metabolism, including insulin resistance and mitochondrial dysfunction, serves as a central driver of sarcopenia (108). This inflammation-energy metabolism axis actively impairs anabolic pathways such as PI3 K/AKT/mTOR, highlighting the potential for pro-metabolic and anti-inflammatory combination therapies to disrupt this catabolic cycle (108). Furthermore, specific metabolic agents have been heavily investigated for their capacity to improve skeletal muscle mass and protein balance. Promising targeted metabolic treatments include beta-hydroxy beta-methylbutyrate (HMB), leucine, citrulline malate, ornithine, creatine, isoflavones, and vitamin D supplementation (109, 110). While these metabolic modulators have been primarily studied in geriatric and osteoporotic populations, they offer a compelling biological rationale for counteracting the profound metabolic stress and muscle wasting associated with breast cancer therapies (109, 110).

## Challenges and future directions

Despite growing recognition, the field faces critical challenges in both definition and biology. Diagnostic heterogeneity across EWGSOP2, GLIS, and AWGS 2025 criteria, compounded by variable imaging modalities and ethnicity-specific thresholds, limits cross-trial comparability and clinical generalizability. There remains an unmet need for consensus definitions that explicitly differentiate geriatric sarcopenia from cancer treatment–emergent muscle wasting, alongside standardized perioperative assessment protocols. Concurrently, the molecular dialogue between breast cancer subtypes and skeletal muscle homeostasis is poorly understood; while systemic inflammation and proteasomal degradation are established drivers, subtype-specific mechanisms remain obscure, and the metabolic determinants of myosteatosis as an early biomarker of muscle quality await elucidation through integrated multi-omics approaches.

Looking forward, robust interventional evidence and pragmatic implementation must advance in parallel. Breast cancer–specific randomized trials are needed to define the optimal timing, intensity, and composition of exercise–nutrition prehabilitation, and to validate emerging pharmacological targets such as the GDF-15–GFRAL and myostatin pathways in combination with standard cytotoxic therapy. Equally important is the development of scalable, tiered screening cascades, from bedside ultrasound and wearable monitors to composite biomarkers integrating muscle, inflammatory, and nutritional indices, that can be embedded within multidisciplinary breast cancer pathways. Only through such standardized, prospective integration can muscle health be elevated from a prognostic biomarker to a genuinely modifiable therapeutic target across the continuum of breast cancer care.

## Conclusion

Sarcopenia is a highly prevalent and clinically consequential syndrome in breast cancer, driven by the combined effects of tumor-induced systemic inflammation, surgical trauma, and the cytotoxicity of anticancer therapies. Rather than acting as a mere passive reflection of aging or advanced disease, skeletal muscle depletion operates as a robust, independent predictor of adverse oncological outcomes. Low muscle mass consistently forecasts inferior overall and disease-free survival, reduced rates of pathological complete response, heightened chemotherapy toxicity, and an increased risk of postoperative complications. Despite these clear associations, clinical translation is currently hindered by heterogeneous diagnostic criteria and the lack of standardized screening protocols. To overcome this, incorporating pragmatic, validated body composition assessments into routine oncological care is essential for early risk stratification. Most importantly, sarcopenia is a modifiable host factor. Early integration of multimodal interventions, specifically structured resistance exercise paired with targeted nutritional optimization, demonstrates strong potential to mitigate muscle wasting and enhance physiological resilience. The primary take-home message is that skeletal muscle health must be elevated from an observational prognostic biomarker to an active therapeutic target. Future priorities must include standardized diagnostic consensus and prospective, breast cancer-specific clinical trials to validate both lifestyle prehabilitation and emerging pharmacological strategies, ultimately improving treatment tolerance and long-term survivorship in this vulnerable population.
